# Dopaminergic novelty detection and theta oscillations: Virtual reality‐based adaptive interventions for cognitive enhancement in aging

**DOI:** 10.1002/ibra.70021

**Published:** 2026-05-24

**Authors:** Abraham Olufemi Asuku, Ayodeji Johnson Ajibare

**Affiliations:** ^1^ Department of Medical Biotechnology, Bioresources Development Centre National Biotechnology Research and Development Agency Ogbomoso Nigeria; ^2^ Department of Physiology, Faculty of Basic Medical Sciences Lead City University Ibadan Nigeria

**Keywords:** cognitive decline, dopamine, electrophysiology, environment, hippocampus

## Abstract

Aging closely correlates with impaired cognitive abilities, including learning, attention, and memory. These impairments are typically linked to disturbances in dopaminergic signaling and hippocampus theta oscillations, two essential processes involved in memory encoding and novelty detection. This review examines the relationship between theta oscillation activity and dopaminergic novelty detection processes, offering a fresh framework for improving adult cognitive function through virtual reality (VR)‐based methods. The specific purpose of this review is to investigate how dopaminergic novelty detection and theta oscillations can be leveraged through VR‐based adaptive interventions to enhance cognitive function in aging populations. It investigates how exposure to new stimuli in a VR setting may activate dopaminergic neurons and induce synchronized theta rhythms to improve memory and learning. The potential for adaptive VR systems that adjust task difficulty and novelty based on real‐time neural feedback is also clarified by this review. By combining results from behavioral, electrophysiological, and neuroimaging research, it supports the development of tailored, non‐invasive therapies that focus on the neurological underpinnings of cognitive decline. This approach is promising for reducing age‐related cognitive decline and enhancing cognitive resilience throughout life.

## Graphical abstract

Aging‐related cognitive decline is associated with reduced dopaminergic signaling and disrupted theta oscillations, which together impair novelty detection and memory formation. This review shows how VR environments can be used to deliver adaptive, novelty‐rich stimuli that engage dopaminergic circuits and entrain theta rhythms, thereby enhancing hippocampal–prefrontal network function. By dynamically tailoring task complexity and sensory input, VR‐based interventions promote neuroplasticity, improve attention and learning, and offer a promising, non‐invasive strategy for cognitive enhancement in aging populations.

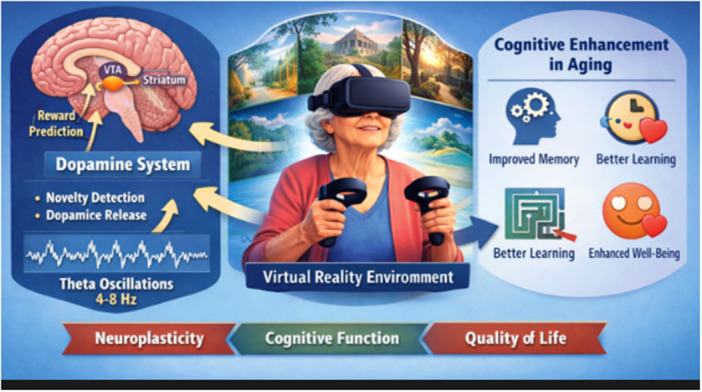

## 1 INTRODUCTION

As people age, cognitive performance becomes increasingly important for functional independence. Furthermore, human communication, such as sensory data processing and integration, as well as reacting to others correctly, depends on intact cognition. As people age, their cognitive capacities tend to deteriorate. It's critical to recognize the kinds of cognitive changes that are typical of aging and those that could indicate the start of a brain disorder. Given the sharp rise in the number of persons over 65 and the rising incidence of age‐related neurodegenerative dementias, it is critical to comprehend how aging affects cognition.

According to Jongsiriyanyong and Limpawattana,^1^ there is a broad spectrum of cognitive decline in the elderly, ranging from mild cognitive impairment (MCI) and dementia to normal cognitive decline with age. Memory loss is a hallmark of MCI, which lies on the boundary between dementia and normal aging.^2^ MCI is the early stage of memory loss or other cognitive skill loss (such as language or visual/spatial perception) in people who are still able to do independent daily tasks.^3^ According to Muhammad et al.,^4^ people with moderate cognitive impairment typically undergo cognitive deterioration with little impairment in their ability to do daily tasks.

Memory, attention, executive cognitive function, visuospatial, and language skills are among the distinct cognitive domains into which cognitive talents can be subdivided. Age‐related reductions are detectable in each of these categories.^5^ Age‐related declines in sensory perception and processing speed affect test scores across a variety of cognitive domains. While some parts of memory remain constant as people age normally, new learning skills consistently deteriorate with age, and retrieval of newly learned material also declines.^5^ Age causes deterioration in prospective memory, specifically the ability to recall and to carry out future planned actions.

It may come as no surprise that earlier empirical research found that novelty can enhance memory because it is a biologically meaningful signal.^6^ Numerous studies have examined the positive effects of novelty on memory in animals. Animals are frequently placed in unfamiliar environments to manipulate novelty in their research (e.g., a new cage with unknown elements [a novel condition] instead of the home cage [a familiar condition]). This kind of novelty has been called environmental or spatial novelty. The presentation of task‐irrelevant novelty usually results in distraction from ongoing activities, according to previous human studies on stimulus novelty (e.g., including novel sights, sounds, or vibrotactile stimuli).^7^, ^8^

The hippocampus releases dopamine in response to novel events, which helps people remember things for longer. Recent research has shown that noradrenergic neurons in the locus coeruleus (LC) corelease noradrenaline and dopamine in the hippocampus, and that their dopamine release also improves memory retention. Previously, it was believed that axons from the ventral tegmental area (VTA) were the only source of dopamine in the hippocampus. Through systems memory consolidation, novel events that have some similarities to those from the past (referred to as “common novelty”) stimulate the VTA and encourage the establishment of semantic memory. On the other hand, “distinct novelty” experiences—those that have little to do with prior experiences—activate the LC to initiate strong initial memory consolidation in the hippocampus, leading to vivid and enduring episodic memories.^9^ Continuous electroencephalography (EEG) signals are transformed from the frequency to the time domains and separated into bands with Greek letter designations (beta, delta, alpha, theta, and gamma) in order to describe oscillatory neural activity. One of the most noticeable frequency bands in the EEG power spectrum is the theta band, which has an intriguing paradox: higher theta power during cognitive tasks is linked to better cognitive performance, whereas elevated theta power during resting states is linked to lower cognitive abilities in children and adolescents.^10^

Computer‐based programs and paper‐and‐pencil exercises have been the mainstays of traditional cognitive rehabilitation techniques, but they might not provide the engagement and real‐world applicability required for successful recovery. Virtual reality (VR) has been receiving increasing attention as a transformational tool in the fields of neuroscience, neuropsychology, and cognitive rehabilitation.^11^, ^12^ VR has the potential to fundamentally alter the approach to cognitive rehabilitation by immersing users in interactive, multisensory environments.^11^ It also offers new opportunities to enhance cognitive functions, promote neural plasticity, and ultimately improve the lives of individuals with cognitive impairments.^13^ This review explores the role of dopaminergic system and theta oscillatory activity of the brain in detecting novelty, which is one of the critical mechanisms by which learning and memory take place. It uses the potential of immersive VR environments to investigate adaptive interventions that can induce these neural processes to improve cognitive functioning in older adults. It connects the basic neuroscience and practical technology by exploring the condition of how specific VR experiences can regulate dopaminergic signaling and theta rhythms, which will eventually offer an alternative approach to cognitive enhancement among aging populations.

## 2 DOPAMINERGIC MECHANISMS IN NOVELTY DETECTION

### 2.1 Neurobiology of novelty detection

Numerous studies have examined the roles of the amygdala and the medial temporal lobes (MTL) structures, which include the entorhinal (ERC), perirhinal (PRC), and parahippocampal (PHC) cortices and the hippocampus, in relation to familiarity, remembrance, and, in a few instances, novelty. It is obvious from the serious memory loss that ensues after damage to the hippocampus, that the hippocampus is an essential part of memory within this web of interconnected parts. The findings demonstrate that, albeit performing comparatively different roles within this network, the MTL structures collaborate with one another^14^ and with an extended brain network^15^ to facilitate novelty detection, memory encoding, and retrieval. In addition to highlighting its evolutionary value, novelty detection triggers a series of behavioral and neurological reactions that allow for flexible memory storing and exploration of the novel information. However, when frequent exposure to novelty leads to rapid neuronal adaptation across the novelty network, this novelty response quickly fades.^16^ Accordingly, novelty detection is linked to a number of different but connected processes, each of which has a specific function. These processes include the mismatch signals production, stimulus initial assessment, and the unexpected results monitoring when applicable, novel stimuli incorporation into pre‐existing ones, and ultimately the new representations development.^6^ Although each of these processes is essential for detecting novelties, little is known about their precise roles or neurological underpinnings. As a result, novelty refers to a quality that we can ascribe to a stimulus (no matter how complicated) in the absence of an existing representation. However, the nature of the pre‐existing representations distinguishes and determines the origins, or sorts, of innovation. Therefore, a novel stimulus is the one that has never been experienced before; the novelty pertains to the stimulus in this instance. To distinguish it from contextual novelty, we will refer to this as absolute novelty.

Numerous studies have examined the function of the hippocampus and nearby PRC in novelty detection.^6^ Nevertheless, a network of brain areas whose functional relevance is still poorly understood is involved in novelty detection. Crucially, it has not been thoroughly examined to what extent the hippocampus and other brain regions that take part in novelty detection may react differently to various kinds of novelty.^17^ Furthermore, it has been largely ignored by recognition memory models and its neural underpinnings that novelty might not be just “no familiarity,” but rather that familiarity and novelty might be two non‐dependent processes that contribute differently to the decisions of recognition memory.

The MTL cortical network that underpins familiarity memory includes the mediodorsal thalamic nucleus (MDt). Crucially, new research indicates that the MTL area may play a role that is independent of material, which is the kind of cuing stimulus, demonstrating functional connection between material‐specific regions in the PRC and PHC while familiarity decisions are being processed.^14^

Although research has shown that the anterior hippocampus plays a role in novelty detection,^18^, ^19^ its functional importance is yet unknown. Weak novelty is associated with much stronger activity than weak familiarity, suggesting that the anterior hippocampus is wired to distinguish between familiarity and novelty responses at low confidence levels.^15^ This pattern points to the quick activation of a familiarity and novelty discriminating mechanism, where the anterior hippocampus—even in weak cases—is essential for identifying novelty.

According to most research on novelty, the anterior parts of the hippocampus and the parahippocampal gyrus—particularly the PRC—are involved in novelty detection.

**Figure 1:**
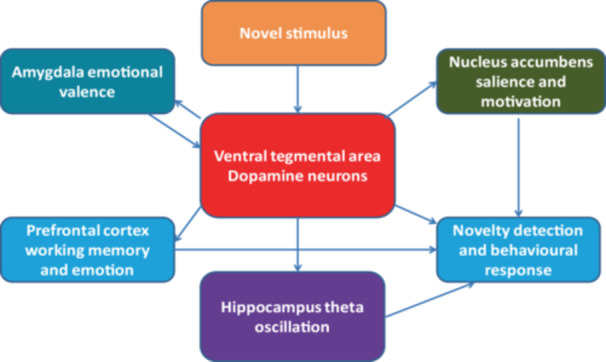
Multiple mechanisms by which novel stimuli engage the VTA dopamine neurons, hippocampus, prefrontal cortex, amygdala and nucleus accumbens to enhance salience, learning, and memory. Novelty detection engages VTA dopamine neurons, leading to phasic dopamine release that modulates hippocampal plasticity, enhances prefrontal cognitive processing, and influences amygdala‐mediated emotional salience. Concurrently, dopaminergic projections to the nucleus accumbens facilitate reward‐related learning and motivational salience. Together, these coordinated circuit‐level interactions promote enhanced encoding, consolidation, and adaptive behavioral responses to novel experiences.

The prevalent belief that the hippocampus and PRC play remarkable and different roles in recognition memory judgments may seem to be contradicted by this constant trend, which indicates that the two areas are novelty network parts. But as was already mentioned, novelty can manifest itself in a variety of ways; therefore, it's likely that multiple mechanisms are involved in its detection (much like in the detection of familiarity) as shown in Figure 1. Furthermore, the anterior hippocampus and the PRC have comparable connection profiles, which put them in a great position to cooperate while also possibly contributing differently to memory in response to novelty detection. Figure 1 below shows the multiple mechanisms involved in novelty detection.

### 2.2 The dopaminergic projections of the substantia nigra and ventral tegmental area

Contrasting predicted absolute novelty with contextual novelty revealed robust activation of the substantia nigra/ventral tegmental area (SN/VTA). This clearly implies that the expected or unexpected stimuli nature in the present context is what causes the novelty that is associated with the dopaminergic modulation of the hippocampus. Thus, for novel stimuli of specific salience—for instance, contextual novelty, unexpected information, or a specific rewarding stimulus—the novelty signal in the anterior hippocampus^15^ is sent to SN/VTA. Integrating the information that is presented with what is already known is crucial since all of these stimuli have one thing in common: they are motivational and have the ability to direct future behavior. The two‐way linkages that define the hippocampus‐midbrain circuit enable this integration. The midbrain releases of dopamine, and more especially the SN/VTA, meet at the hippocampus, causing long‐term potentiation (LTP) and opening the door to new learning, according to the well‐known model put forward by Lisman & Grace.^20^ In the instance of the detection of novelty, this process is responsible during the detection of contextual novelty. As previously mentioned, it seems to be specialized for motivationally significant stimuli. Because the improved connection of SN and VTA entails the posterior and anterior features, the resulting memory encoding is probably involving the posterior and anterior hippocampus.^15^ This explains recently documented discrepancies concerning the selectivity of the anterior hippocampus' function in novelty detection/encoding^21^ and is in line with the evidence corroborating the roles of both posterior and anterior hippocampus in encoding.^22^

Thus, only the anterior part of the hippocampus plays a role in the novelty initial detection (contextual or absolute), but after midbrain/striatal dopaminergic stimulation, both the anterior and posterior hippocampuses encode novel information.

A functional loop is formed between the VTA and hippocampus dopaminergic neurons in the midbrain. When the hippocampus picks up information that hasn't been stored in its long‐term memory yet, the loop is activated. Via the accumbens, subiculum, and ventral pallidum, the resulting novelty signal is transmitted to the VTA, where it helps these cells fire in a novelty‐dependent manner together with salience and goal information. Dopamine is released in the hippocampus in the upward arm of the loop, which improves learning and LTP. These results provide credence to a theory that the hippocampal‐VTA loop controls how information enters long‐term memory.

Both dopamine synthesis and receptors alter with aging, which impacts cognitive performance; synthesis tends to rise while receptors tend to fall.^23^

The dopaminergic loop experiences a progressive functional impairment, indicating a decrease in dopamine production, availability of receptors, and their signaling power, in the aging process. These alterations lead to age‐associated deterioration of motor coordination, executive functioning, working memory, as well as sensitivity of rewards and heighten susceptibility to neurodegenerative diseases like Parkinson's disease. Therefore, the dopaminergic loop is imperative to the cognitive and motor resilience in aging and is a primary focus of interventions used to boost healthy aging of the brain.

## 3 THETA OSCILLATIONS IN LEARNING AND MEMORY

### 3.1 Biophysical properties of theta oscillations

Theta oscillations are low‐frequency brain rhythms (4–8 Hz in human beings, 6–10 Hz in rodents) that are formed through the combined actions of neuronal membrane properties, synapses, and network structure. At the cellular level, theta rhythms in the brain are facilitated by intrinsic resonance characteristics of the neurons, specifically the hippocampal and the entorhinal pyramidal cells. Membrane resonance in the theta range is provided by voltage‐gated ion channels and low‐threshold calcium currents. Based on these currents, neurons are able to selectively respond to inputs at theta frequencies.

The theta oscillations depend on the regulation of excitatory glutamatergic transmission and inhibitory GABAergic transmission synaptically. Rhythmic inhibition of pyramidal cells by fast‐spiking interneurons, especially those expressing parvalbumin and by somatostatin‐expressing interneurons, provides phasic inhibition that precisely times the firing of pyramidal neurons, thereby shaping the timing and synchronization of theta oscillations.

Recurring excitation–inhibition loops within circuits in the hippocampus are produced at the network level, as well as long‐range inputs produced by the medial septum, a pacemaker. Septal cholinergic and GABAergic tract extends and controls neuronal excitability and theta across distributed parts of the brain. The biophysical characteristics of theta oscillations are a result of the interaction of intrinsic membrane resonance, synaptic time constants and network synchronization that allows theta to synchronize neurons' firing on a behaviorally relevant timescale to cognitive and memory.

### 3.2 Role of theta oscillations in encoding and retrieval of memory

The theta activity during encoding of memories helps in organizing the incoming information through a temporal structure of neuronal firing in order to augment the synaptic plasticity processes like LTP. High theta and phase correspondence between the hippocampus and the prefrontal or entorhinal cortices are always related to the process of successfully encoding new information. Inter‐communication and processing in these parts, as well as with the association areas of the neocortex, have been connected with both spatial navigation and episodic memory.^24^ These processes involve links between the cognitive and sensory components that consist of an event memory or between the traits that identify certain locations in space. Furthermore, these processes might be dependent on the theta rhythm. It has been discovered that during theta phase, MTL neurons that represent specific geographical locations are activated. This system's continuous theta rhythm allows data to be encoded in terms of firing rates and spike‐phase relationships, providing a trustworthy standard for distributed cellular activity. Furthermore, the systematic co‐activation of progressively visited regions enables spike‐timing dependent plasticity (STDP), which reinforces connections between sequentially activated cells with a bias for forward‐associations. Although it need not be limited to spatial memory, this process might be essential for establishing temporal associations between random inputs. Indeed, it is associated with two of the primary features of episodic free recall: the ability to recall events that occurred in close succession, and forward‐asymmetry, or a preference for forward transitions.^25^

The theta oscillation promotes the reactivation and incorporation of stored memory traces in retrieving memory. It is believed that the theta phase coherence within distributed brain networks oversees the reinstatement of contextual and associative information. Significantly, the separation of encoding and retrieval processes can be achieved by the timing of neuronal firing with respect to the theta phase (theta phase coding), allowing more information to be stored and retrieved with less interference between new and stored information.

### 3.3 Theta‐gamma coupling

The theta‐gamma coupling (TGC) is a type of cross‐frequency coupling where the amplitude or timing of the faster gamma oscillations (about 30–100 Hz) in the brain is modulated by the phase of slower theta oscillations (about 4–8 Hz). It is best seen in the hippocampus and neocortex, and is regarded as a basic process of neural information processing.

The TGC is believed to have a functional purpose in facilitating working memory, episodic memory, learning, and attention by arranging neuronal firing into windows of time. A number of gamma cycles may happen in a single theta cycle, and each of them may represent a different piece of information, so‐called neural code, in the case of sequential memory coding.

TGC disruption has been associated with cognitive impairment, neuropsychiatric, and neurodegenerative diseases such as the Alzheimer's, schizophrenia, and Parkinson's diseases. In this regard, TGC is becoming a biomarker of cognitive performance as well as a possible neuromodulatory intervention target, including brain stimulation and neurofeedback.

### 3.4 Changes of theta oscillations in aging

Various typical changes in theta activity in the rest and task state are linked to aging.

According to Perone et al.,^26^ theta decreased with age and children and adolescents with higher theta power during resting‐state recordings had lower cognitive capacity, indicating a negative relationship between the two variables. However, theta power and behavioral performance during cognitive tasks have been regularly demonstrated to be positively correlated in task‐related EEG research.^27^ Slower oscillations like delta and theta dominate the resting EEG power spectrum in young children, with sustained oscillations mostly absent in infant.^28^

Resting‐state theta power usually slightly increases in some regions of the brain during healthy aging, especially in the frontal regions. This growth is often interpreted to indicate less efficient utilization of neural resources or recruitment of new neural resources in order to sustain cognitive functioning.

In cognitive tasks, particularly those that use working memory, novelty processing and episodic memory, the theta power of the task and the synchronization of the theta of older adults are usually less than those of younger adults. This decrease is associated with age‐related memory encoding and executive dysfunctions.

Theta coherence and connectivity are also reduced with aging, and there is a reduction in long‐range frontal theta and frontoparietal theta coupling. These interruptions damage the communication between the brain areas that play important roles in memory and cognitive control. Besides that, theta phase amplitude coupling, especially theta phase gamma amplitude coupling, tends to be reduced in elderly subjects, which implies that they cannot coordinate the cross‐frequency activity to process information effectively.

In general, the age‐related variations in theta oscillations can be discussed as the combination of functional degradation and neuroadaptive compensation, and they have been discussed as the sensitive electrophysiological indicators of cognitive aging and the initial neurodegenerative activity.

### 3.5 Dopaminergic modulation of hippocampal theta oscillations

According to Jamali et al.,^29^ dopamine is one of the neurotransmitters and neuromodulators that influence hippocampus theta activity. Previous studies have demonstrated the role of dopamine receptor‐2 signaling in regulating hippocampal rhythms. Increased extracellular dopamine lowers the frequency of hippocampus theta oscillations caused by secondary alterations in the serotonergic neuromodulatory system.^30^ The hippocampus theta rhythm power was enhanced by an intraventricular infusion of a dopamine reuptake blocker, which also boosted the firing rate and modulation of medial septal neurons.^31^ Orzeł‐Gryglewska et al.^31^ state that theta frequency and power may be affected by the activation of glutamatergic receptors or a decrease in GABAergic tone in the VTA, which typically leads to an increase in dopamine release into the hippocampus.

## 4 VR‐BASED COGNITIVE INTERVENTIONS

### 4.1 VR in spatial navigation and hippocampal function

VR has emerged as a helpful and affordable tool in therapeutic settings, offering safe and entertaining environments for exercising motor and cognitive abilities.^32^ It has many benefits over traditional physical and cognitive therapies, like many‐module flexibility, enhanced ecological validity, and increased intervention compliance.

In order to improve the performance of cognitive processes in VR settings, interventions must entail active participant interaction, and manipulation of environmental factors, including sense of presence, emotional engagement, image quality, and the congruent object/environmental schemas of the presented stimuli to improve recall of objects.^33^ VR interventions have been widely used to improve cognitive functions and physical performances in older persons with moderate cognitive impairment^34^ and healthy cognition,^35^ in addition to improving memory. Autonomy and survival depend on the capacity to navigate different situations and locales from memory. Position and orientation are continuously updated for efficient navigation by combining memory representations of the surroundings with multisensory data.^36^ Visual, vestibular, and proprioceptive input are examples of pertinent sensory data.^37^ These inputs are aggregated and transformed into spatial representations based on real behavioral needs, which are further encoded, consolidated, and finally retrieved.^38^ One important part of the brain networks involved in spatial navigation is the hippocampus. The hippocampus binds and integrates location‐specific information from several sensory modalities to transform spatial relationships into a global cognitive map.^39^ As this map is being created, the hippocampus is continuously interacting with other regions of the brain, sharing computations with the parahippocampal cortex, entorhinal cortex, and retrosplenial cortex, among others.^40^

Therefore, behavioral assessment of spatial navigation has become an important way to assess hippocampal function in different animals. In animal models of spatial memory, particularly in rats, navigation in environments such as the Morris Water Maze (MWM) is a widely used standard.^41^

The absence of body‐based sensory inputs creates an artificial environment that limits the amount of spatial information available for navigation and may lead to behaviors that don't necessarily align with what humans need in their daily lives.^42^

The ecological feasibility of fixed navigation paradigms has thus been hotly debated.^43^ Thanks to advancements in mobile immersive VR technologies, these limitations can be overcome and human spatial navigation can be investigated with full multimodal input.^44^

According to Moreno‐Casilla,^45^ novelty causes the hippocampus to release dopamine, and when exposed to a novel stimuli, dopamine‐releasing tyrosine hydroxylase positive (TH+) neurons in both the VTA and the LC become more active.^46^ Additionally, new experiences support memory persistence,^47^ hippocampus reactivation,^48^ and hippocampus remodeling.^49^

However, novelty can be seen as a spectrum with a variety of flavors. On one extreme of the range are new experiences that have elements in common with previous ones. “Common novelty” is the term for this kind of novelty, and it refers to a new event that is similar to and relevant to previous experiences, and as such, it can be recalled by updating the memories already stored in the neocortex. However, completely new events pose unique challenges to the brain's memory systems.

**Figure 2:**
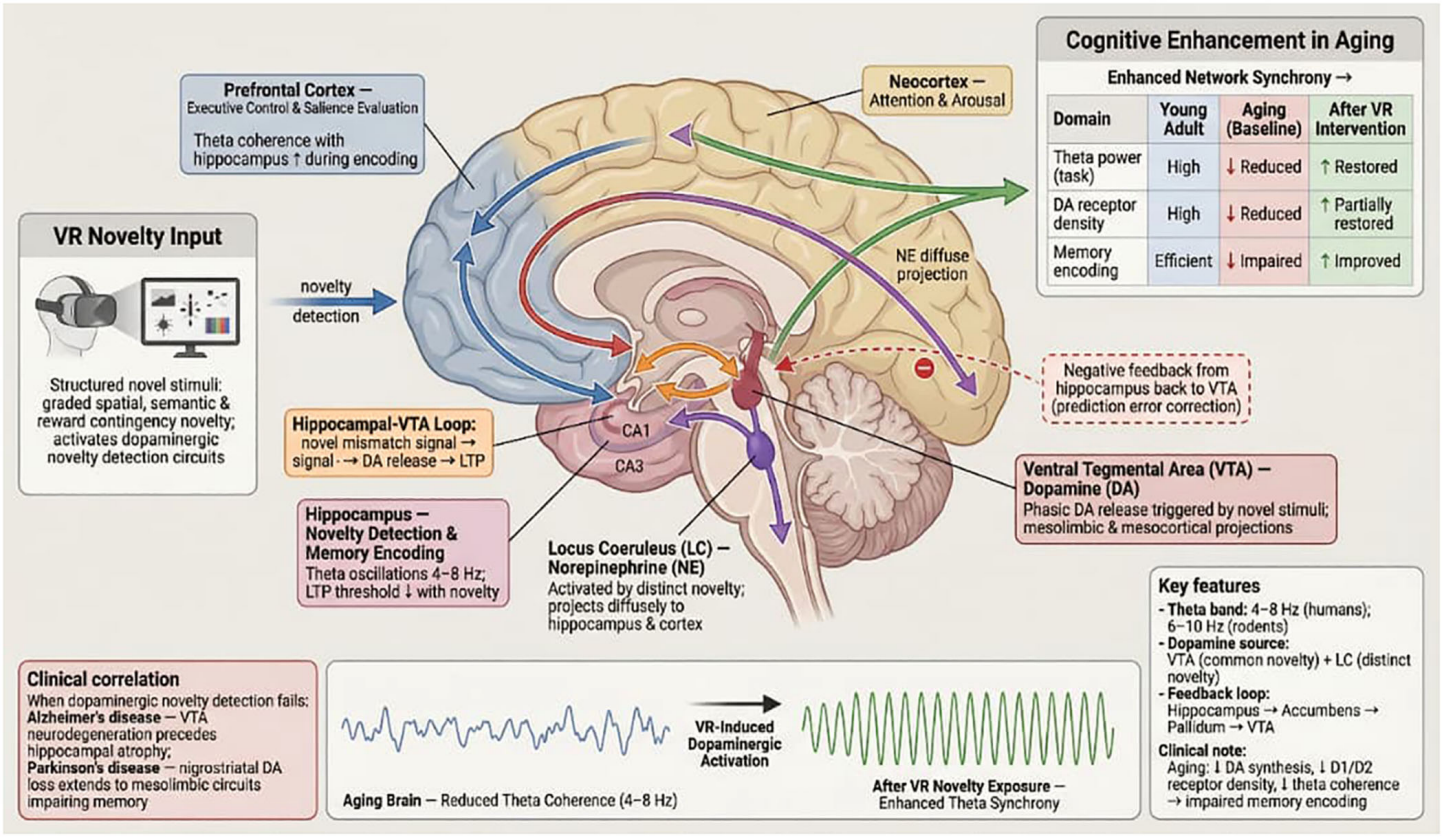
LC‐VTA neuromodulatory pathways supporting dopaminergic novelty detection and theta oscillations during VR‐based cognitive interventions in aging. A sagittal brain illustration showing how VR‐delivered novelty activates the dopaminergic (VTA) and noradrenergic (LC) systems to enhance hippocampal theta oscillations and memory encoding. Color‐coded pathways trace the bidirectional Hippocampal‐VTA loop, the LC's diffuse norepinephrine projection, and VTA's mesolimbic/mesocortical dopamine output. A theta waveform strip at the bottom contrasts the aging brain's reduced coherence with the restored synchrony following VR exposure. A comparison table quantifies the effect across three domains (theta power, DA receptor density, memory encoding), and a clinical correlation box links dopaminergic failure to Alzheimer's and Parkinson's disease.

Such innovation is unique by definition and differs greatly from past encounters. It is therefore less suitable for integration into memory representations already stored in the neocortex and may potentially clash with previously acquired information. Figure 2 below shows the LC‐VTA neuromodulatory pathways supporting dopaminergic novelty detection and theta oscillations during VR‐based cognitive interventions in aging. And Table 1 shows the VR cognitive studies and the main outcomes.

**Table 1:** VR cognitive intervention studies.

Study (design/population)	Intervention	Duration	Cognitive measures	Main outcomes
Tariq et al.^50^	VR‐based cognitive rehabilitation (CR) vs traditional CR	30‐min session 3 times weekly for 12 weeks	MoCA, Trail Making Test, Digit Span	VR group showed significant gains in global cognition and attention/executive scores versus control
– RCT with stroke survivors				
Torpil et al.^51^	VR CR + conventional CR vs CR alone	12 weeks	LOTCA‐G (multi‐domain cognition)	VR enhanced orientation, visuospatial perception, attention
– RCT in older adults with MCI				
Park et al.^52^	VR CR vs conventional CR	30 min per day, 5 days/week, for 6 weeks	MoCA, TMT‐A/B, Digit Span	Greater improvement in MoCA and attention/executive tests in VR group
– RCT *VRCMR* in community MCI				
Kwan et al.^53^	VR motor‐cognitive training vs usual care	16 sessions with one‐hour training twice per week for 8 weeks	Global cognition (MoCA), executive	VR group improved cognitive scores and reduced frailty versus control
– Multicenter RCT				
Thapa et al.^54^	VR game‐based cognitive training	Three times weekly, 100 min, for 8 weeks	MMSE, TMT, SDST + EEG	Improvements in attention, processing speed; neural markers showed favorable changes
– RCT VR + cognitive games in MCI				
Du et al.^55^	Aggregated VR interventions (CR training, exergames, telerehabilitation)	Various RCTs	Composite cognitive outcomes	Moderate effect of VR on cognition across disorders; stronger for rehab/exergames
– Meta‐analysis (Neuropsychiatric disorders)				
Yang et al.^56^	VR training (immersive/non)	Various	MMSE, attention/executive metrics	Small to moderate improvements in attention/executive function
– Meta‐analysis in MCI				

*Notes*:
The table includes RCTs and meta‐analyses with cognitive outcomes, covering stroke, MCI, cognitive frailty, and neuropsychiatric populations. Cognitive outcome measures vary (e.g., MoCA, MMSE, TMT, Digit Span), so effect interpretations should consider domain specificity. Meta‐analytic data show that VR effects are moderate overall but may vary based on intervention type, immersion level, and target domain.

Abbreviations: CR, cognitive rehabilitation; EEG, electroencephalography; LOTCA‐G, Loewenstein Occupational Therapy Cognitive Assessment – Geriatric version; MCI, mild cognitive impairment; MoCA, Montreal Cognitive Assessment; MMSE, Mini‐Mental State Examination; RCT, randomized controlled trial; SDST, Symbol Digit Substitution Test; TMT, Trail Making Test; TMT‐A/B, Trail Making Test Parts A and B; VR, virtual reality; VRCMR, virtual reality‐based cognitive‐motor rehabilitation.

### 4.2 VR, novelty, and theta

VR has swiftly been utilized as an experimental instrument that is amenable to the investigation of novelty processing in part due to its capacity to allow manipulations of spatial, location, and occasion novelty to be in an immersive, controllable way, and due to the potential of neurophysiological activity being recorded simultaneously. Experiments on trans‐lateral activation revealed that theta of the human hippocampal is activated during virtual navigation test on the basis of similarities to rodent theta of space showing VR a valid proxy of hippocampo‐theta communication under noninvasive, noninvasively‐invasive or intracranial recording conditions.^57^

The same impact of novelty, as the parameter measured by novel locations, unexpected events, or prediction errors, on low‐frequency oscillations has a uniformly modulating impact on scalp EEG and intracranial VR experiments. The self‐driven search in the VR also results into an increased midline/fronto‐midline theta compared to the phenomenon when the same 2D screen functions are performed, which indicates that sensorimotor contingencies and embodiment contribute to novelty related to the theta. They are supported by the recently conducted reviews of the EEG + VR literature and empirical findings that suggest that bigger induced theta, P3‐like results to new stimuli are created during immersive experience.^58^

On a mechanistic level, however, time‐dependent theta oscillations of the hippocampus and the medial prefrontal cortex (mPFC) and ventral hippocampus have recently been reported to be involved in processing novelty and offering cues of prediction error. Encoding of novel scenes with the help of mnemonics and encoding of similar inputs with the help of successful discrimination (pattern separation) have been associated with the theta power and the ability to encode novel information as a co‐ordination rhythm.^59^

The application of VR‐EEG paradigms has proliferated in recent years, including spatial navigation tasks (mazes and wayfinding in virtual environments), environmental oddball designs (new object/events in an existing environment), prediction‐violation paradigms (omitted rewards or unexpected threats), and comparisons between active and passive exploration. These studies can also inform the development of adaptive VR systems—for instance, through real‐time modulation of visual complexity based on EEG signals—and facilitate the translation of these research questions to aging populations and clinical patients. In such groups, theta responsiveness to novelty has been shown to predict memory performance and gains from cognitive training.^60^

## 5 REAL‐TIME MONITORING OF NEURAL ACTIVITY AND EEG‐BASED THETA TRACKING FOR INTERVENTION PERSONALIZATION

EEG is a common technique for assessing the activity of the brain in developmental studies. In comparison to other neuroimaging methods like functional magnetic resonance imaging, EEG provides special insights into the dynamics of the brain cognitive functions and is a more accessible technique for measuring the brain's electrical activity. EEG seems to be one of the most promising technologies since it can provide a plethora of information on a subject's mental state.

Research employing magnetic resonance imaging (MRI) demonstrated that one of the factors influencing the impact of institutional rising on resting EEG power was a reduction in cerebral white matter level.^61^ Children from low‐income (as opposed to higher‐income) families^62^ and the ones who experience greater levels of stress from caregiver^63^ have shown similar sequence of the activity of resting EEG. Crucially, randomized control treatments that focus on environmental factors seem to be able to alter these patterns of brain activity.^64^

EEG research related to tasks look at how the brain changes during particular cognitive processes.^65^ Task‐related EEG research regularly revealed favorable connections between theta power and cognitive performance, in contrast to resting EEG research, which has primarily identified negative correlations between theta power and cognitive functioning.

Research on rodents has provided ample evidence of the connections between theta activity and memory functions.^66^ LTP, an increase in synaptic plasticity, is triggered at the cellular level by activating the cells of the hippocampus at the theta oscillations peak. This process is thought to be a crucial cellular mechanism for memory formation. It is also believed that the hippocampal theta plays a role in the transfer of information across different parts of the brain. Better behavioral performance was predicted by elevated theta coupling between the mPFC and hippocampus during spatial memory tests.^67^ All of these investigations on rodents point to the importance of the hippocampus theta in memory functions. Notably, studies on the hippocampus theta in rodents have concentrated on neuronal activity that is directly monitored by implanted electrodes. Human EEG research, on the other hand, has concentrated on the scalp electrical activity, which is not a precise indicator of the activity of the hippocampus. Hence, it is not possible to directly align or generalize the connections between the hippocampus theta and memory processes in mouse study to human EEG research. On the other hand, scalp EEG may offer cortical theta dynamics readout, which may interact with the theta of the hippocampus, as previous studies have shown that the two synchronize.^68^

In fact, there is growing evidence that theta EEG activity measured on the human scalp is also associated with memory functions. In all age groups—adults, children,^69^ as well as adolescents^70^—the EEG power of theta rises at the period of memory encoding and retrieval. Additionally, task performance is predicted by EEG power of theta at the period of tasks involving memory. Better recognition and recall of items were predicted in adults with higher theta power over the parietal and frontal areas, evaluated either before or after encoding of item.^25^ Similar connections have been observed in older children and newborns. Begus and colleagues^71^ investigated 11‐month‐olds' ability to recognize new items using a preferential‐looking task after recording their EEG activity at the time they were investigating the items. They discovered that bigger disparities in infants' later recognition of two things were predicted by bigger differences of theta power over the frontal area between two items at the time of exploration. It was a theta band‐specific effect. In a similar vein, Michel et al.^72^ discovered that higher absolute theta power (3.5–5 Hz) across the fronto‐central region of the brain at the time of object encoding demonstrated improved item identification in infants who are 9 to 10‐month old, utilizing an object exploration/recognition paradigm and a mother/infant contact.

All of these results demonstrate that changes in the EEG activity of theta at the time of item encoding are predictive of later memory performance.

More intricate interactions with gamma oscillations are also involved in the connections between memory processes and theta associated with task. The regulation of the oscillation power of gamma by theta oscillation phase is known as TGC. By offering temporal references for categorizing objects in memory and promoting information transfer across different parts of the brain, this dynamic interaction is believed to support memory function.^73^

The studies on rodents show that the hippocampus theta is essential for memory formation. Elevated TGC and theta power have been seen at the period of memory processing in human scalp‐recorded EEG studies and higher theta inter‐trial phase‐synchrony, theta power, and TGC indicated improved performance of memory process.

## 6 ADAPTIVE VR SYSTEMS FOR COGNITIVE ASSESSMENT

The adaptive VR systems have been established as an effective and efficient instrument in cognitive assessment to some extent of ecological verifiability and test control that is better than paradigms of standard laboratory assessments. Unlike fixed computerized tests, adaptive VR systems are closed‐loop systems, the stimulus and task requirements are dynamically presented in response to the changes in the cognitive state of a participant. By doing so, it is possible to effectively interrogate processes of attention, updating memory, and new‐detection and cognitive control in more realistic situations.

The key concept of adaptive VR assessment is a multimodal monitoring structure that integrates behavioral, physiological, and, in case available, neurophysiological measures on a real‐time basis. Reaction times, task accuracy, efficiency of the trajectory in spatial tasks, and search/exploration patterns, which are all based on motion tracking, are the most widespread behavioral metrics. The physiological measures that provide complementary data about arousal and cognitive load are heart rate variability, galvanic skin response, and pupil dilation. The sensitivity is added to by the neurophysiological indicators, particularly EEG‐based ones. Frontal midline theta of interest (4–7 Hz) has a sound association with the novelty detection, replenishment of the working memory, and cognitive control. Temporary accelerations of theta in VR‐based novelty paradigms could be an indication of successful processing of unexpected events, whereas sustained increases may be indicators of compensatory processing or processing load.

Multimodal signals are computed in a computational decision layer that subsequently continually approximates the mental condition of the user. The machine‐learning algorithms interpolate the information that is received to inform whether the participant is under challenged or is challenged in the right way, or he/she is overchallenged. When this is concluded, the system moderates the amount of novelty and the amount of difficulty in the task of the VR setting. The changes may be the addition of additional objects, modification of the patterns of space, modification of the aspects of the environment (e.g., lighting, sound, or information in the surroundings), or modification of time and predictability in events. To illustrate this, when the answers are slow and accurate, that is the sign of under‐stimulation, and in this case, the system can introduce a more unpredictable or more complex stimulus that maintains attention. Conversely, the increase in the rate of errors as well as physiological signs of strain or a high theta can lead to the system reducing novelty, simplifying the goal, or prolonging the inter‐event interval.

This dynamic adaptation mechanism typically occurs in short‐temporal scales ‐ of a few seconds‐minutes—such that the VR world can be maintained synchronous with the moment‐to‐moment cognitive profile of the subject of interest. This is particularly crucial in situations of heterogeneity in cognitive abilities, which includes aging, mild impaired cognitive persons, or neuropsychiatric and neurodegenerative pathology. Notably, adaptive VR enables the researcher to query in a methodical way on how the brain responds to novelty under controlled naturalistic circumstances, providing data on the nature of learning, attention, and memory mechanisms that would be difficult to assess using fixed tasks.

### 6.1 Proposed adaptive intervention framework for dopaminergic novelty and theta for cognitive enhancement in aging

**Figure 3:**
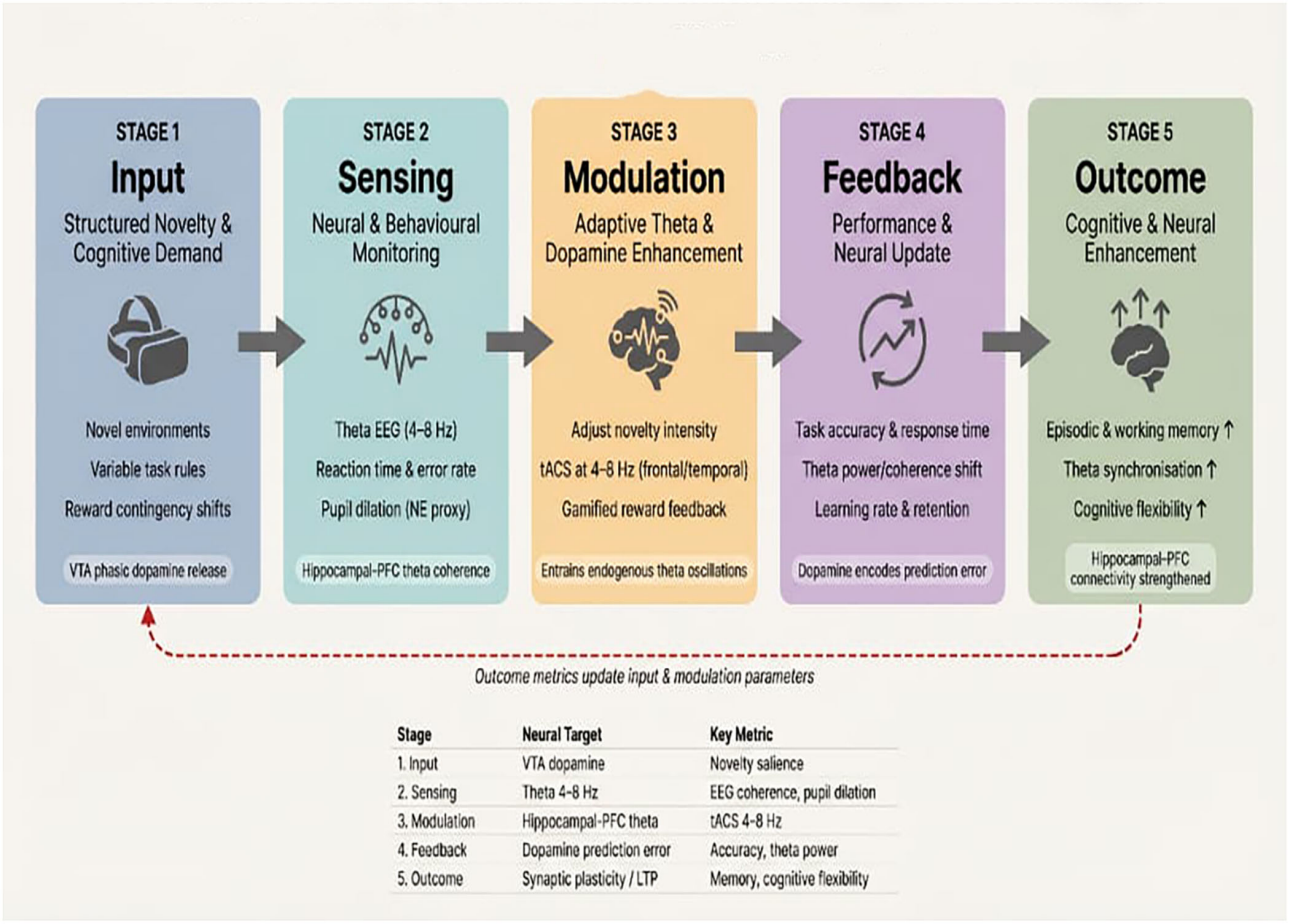
Schematic of the adaptive VR intervention pipeline. The framework comprises five sequential stages: input (structured novelty and cognitive demand), sensing (neural and behavioral state monitoring), modulation (adaptive dopaminergic and theta enhancement), feedback (performance and neural update loop), and outcome (cognitive and neural enhancement).

By combining VR technologies with the neurobiological concepts of theta oscillations and dopaminergic novelty detection, the suggested adaptive intervention framework focuses on a translational system. It offers a promising way to enhance aging resilience, cognitive strength, and plasticity by customizing immersive experiences to individual neural and cognitive circumstances (Figure 3).

### 6.2 Stepwise adaptive intervention model

The framework is organized as a closed‐loop adaptive system with five stages:

1. Input
2. Sensing
3. Modulation
4. Feedback
5. Outcome

Each stage is justified by established neuroscience principles.

#### 6.2.1 Step 1: Input – structured novelty and cognitive demand

##### Description

Participants are exposed to graded novelty stimuli embedded in cognitively demanding tasks. These may include novel learning environments, variable task rules, unexpected reward contingencies, and contextual shifts in sensory or semantic information.

##### Neuroscientific justification

Novelty is a primary driver of phasic dopamine release from the VTA, which projects to the hippocampus and prefrontal cortex (PFC). Novel stimuli enhance hippocampal plasticity by lowering LTP thresholds and increasing memory encoding efficiency.^74^ In aging, externally structured novelty compensates for reduced endogenous exploration and motivational drive, re‐engaging dopaminergic circuits critical for learning.

#### 6.2.2 Step 2: Sensing – neural and behavioral state monitoring

##### Description

Theta‐band activity (EEG/MEG; frontal‐midline and hippocampal proxies), behavioral markers (reaction time variability, error rates), and optional physiological correlates (pupil dilation as a proxy for neuromodulatory arousal) should be monitored real‐time. However, because pupil size in immersive VR is strongly influenced by rapid scene luminance changes, pupillary features should be interpreted only after controlling for luminance (e.g., logging headset/scene brightness and regressing out light‐reflex variance or applying light‐reflex correction) before attributing residual dilation to cognitive/neuromodulatory arousal.^75^, ^76^, ^77^

In practical VR/mobile EEG settings, real‐time theta tracking typically relies on a low‐latency pipeline (streaming bandpass/line‐noise control, short‐window feature extraction, and continuous artifact handling with quality‐gating). For example, Artifact Subspace Reconstruction can suppress transient high‐amplitude artifacts,^78^ and online/recursive ICA approaches can incrementally separate artifact‐related components during ongoing recordings;^79^ these steps can be complemented by rejecting or down‐weighting segments with excessive motion or poor sensor contact before updating the adaptive controller.”

##### Neuroscientific justification

Theta oscillations reflect hippocampal–prefrontal coordination, supporting working memory, episodic encoding, and cognitive control.^80^ Aging is associated with reduced theta coherence and delayed phase synchronization. Continuous sensing allows detection of suboptimal neural states, such as low theta power or reduced engagement, enabling timely intervention before performance deteriorates.

#### 6.2.3 Step 3: Modulation – adaptive dopaminergic and theta enhancement

##### Description

Based on sensed state, the system delivers targeted modulation: which includes cognitive modulation ‐ adjusting novelty intensity, task difficulty, or reward salience, neural modulation ‐ non‐invasive brain stimulation (e.g., theta‐frequency transcranial alternating current stimulation [tACS] over frontal or temporal regions), and behavioral modulation ‐ adaptive feedback, gamified incentives, or motivational cues. In future closed‐loop extensions, the modulation controller could also be guided by cross‐frequency targets (e.g., theta phase–gamma amplitude coupling) rather than theta metrics alone, given evidence that aging is associated with reduced/less precise theta–gamma coupling in older adults.^81^, ^82^

##### Neuroscientific justification

Theta‐frequency stimulation has been shown to entrain endogenous oscillations and improve memory and cognitive control, particularly in older adults. Dopamine‐sensitive learning systems benefit from precision‐tuned reward signals, avoiding overstimulation that could impair performance via inverted‐U effects.^83^ Adaptive modulation aligns intervention strength with current neural capacity, preserving homeostasis while enhancing plasticity.

#### 6.2.4 Step 4: Feedback – performance and neural update loop

##### Description

The system evaluates task performance changes, theta power/coherence shifts, and learning rate and retention. These metrics inform subsequent input and modulation parameters.

##### Neuroscientific justification

Closed‐loop feedback mirrors biological learning systems, where dopamine signals encode prediction error and guide future behavior. Feedback‐driven adaptation is essential in aging, where interindividual variability in neuromodulatory decline is high.^84^ This step ensures personalization and long‐term efficacy rather than fixed‐dose interventions.

#### 6.2.5 Step 5: Outcome – cognitive and neural enhancement

##### Description

Expected outcomes include improved episodic and working memory, enhanced cognitive flexibility, increased theta synchronization, and greater learning persistence and transfer.

##### Neuroscientific justification

Sustained engagement of dopamine–theta interactions promotes systems‐level plasticity, strengthening hippocampal–prefrontal communication and compensatory network recruitment in aging brains.^85^

### 6.3 Feasibility

Current feasibility includes widely available and low‐cost EEG‐based theta monitoring, the existence and possible extension of adaptive cognitive training platforms, and the guaranteed safety of non‐invasive stimulation (tACS/tDCS) in older adults.

### 6.4 Evidence and preliminary findings

Pilot studies show that closed‐loop neurostimulation improves working memory and attention more effectively than open‐loop approaches.^86^

A novel arrangement of the labyrinth walls, the position of the reward, and the orientation of the local and surrounding spatial signals in the crossword maze are all examples of common novelty in rodent trials. Place cell firing measures the global remapping of the hippocampus's cognitive map in this extremely unfamiliar environment.^48^ However, there is a cognitive basis for the retention of the new experience because parts of the activity and the environment have been encountered previously. In these investigations, a retrieval test conducted 1 h later showed that optogenetic activation of hippocampus VTA‐TH+ axons was sufficient to improve memory retention. The initial consolidation of memory in the hippocampus may be triggered by dopaminergic regulation of synaptic plasticity.^87^ Such extra‐hippocampal effects may therefore further contribute to dopamine's effect on systems memory consolidation, as dopamine release in the PFC has been demonstrated to enhance hippocampal‐PFC coherence and subsequent reactivations during sleep.^88^ The components of these new episodes can be integrated into neocortical semantic knowledge structures based on prior experience when the VTA‐hippocampus system is activated by common novelty.^89^ As a result, the memories should become less vividly episodic in quality. Furthermore, this typical novelty‐associated memory boost involving VTA is more specific to the new events themselves than the more noticeable “grace period” linked to dopamine released by the LC‐hippocampal system (which results in a more generalized memory increase).

An experiment in which rats were permitted to investigate previously unobtainable arms of a radial arm maze—representing novel experiences in a familiar setting—provides evidence for this.^90^ Improved reactivation of representations linked to familiar arms visited at about the same time did not coincide with improved reactivation of the novel arm images. According to McNamara,^48^ in crossword maze experiments, the reactivation of the hippocampus was only enhanced for spatial maps created during the optogenetic activation of hippocampus VTA‐TH+ axons; it did not improve reactivation of maps that were already in the hippocampus network before the optogenetic manipulation.

Although distinct novelty can take many different forms—for example, new floor substrates, objects, and spatial context in mouse experiments—it is invariably unrelated to the animal's prior experiences.^91^ The recently described dopaminergic LC‐hippocampus system is activated when animals are presented with a singular, one‐of‐a‐kind experience.^46^ For events that occurred both before and soon after an event that was marked by distinct novelty, dopamine from hippocampus locus coeruleus tyrosine hydroxylase positive (LC‐TH+) afferents produces a “grace period” of enhanced initial memory consolidation. This period provides the rich contextual details that are characteristic of long‐lasting hippocampal‐dependent memories.^46^ The expression of immediate early genes in the PFC following learning is downregulated by such distinct novelty, according to a recent study.^91^ This shows that such novel experiences impede the processes of subsequent systems consolidation. Additionally, different novelty results in a longer retention period for the memory in its detailed, hippocampus form, and its consolidation is independent of sleep, possibly indicating that it is not dependent on hippocampus reactivation.^91^

In particular, there is no data to support the use of VR technology for integrated cognitive and motor training in older persons, particularly in poor nations.^92^, ^93^ Prior research has indicated that cognitive‐based training can help healthy older persons improve their cognitive functioning.^94^, ^95^ A major issue that makes daily tasks difficult and causes significant costs for those who suffer from it is prospective memory impairment, which presents as an inability to recall delayed intents.^96^ By allowing patients to practice prospective memory tasks like making coffee in a virtual kitchen,^97^, ^98^ or making purchases from a shopping list in a virtual convenience store,^99^ non‐immersive VR‐based cognitive rehabilitation programs that operate on a desktop computer enable cost‐effective training of patients. Patients respond favorably to this type of VR‐based training, which has shown promising improvements in cognitive abilities that rely on frontal lobe functions, such as accurate performance of event‐, time‐, and ongoing tasks and instant recall of prospective memory tasks.^100^ It is believed that changes in neural plasticity that improve working memory are linked to notable learning gains after a VR exercise program. Additionally, VR dramatically improves selective attention, cognitive flexibility, and shifting abilities, which improves behavioral outcomes for individuals with brain injuries.^101^ Enhancement of problem‐solving abilities and selective memory processes promotes social reintegration and improves career results.^102^

### 6.5 Technological requirements

The following are needed: high‐density or mobile EEG systems with real‐time processing, adaptive algorithms (machine learning‐based state classification), non‐invasive stimulation devices capable of frequency‐specific entrainment, secure data integration platforms for longitudinal monitoring, and user‐centered design to ensure adherence in older populations.

## 7 POTENTIAL MECHANISMS OF COGNITIVE ENHANCEMENT

The development, updating, and retrieval of memories—the foundation of outcome prediction—are all significantly influenced by the hippocampus. The hippocampus uses long‐term synaptic plasticity, which can be either depression (LTD) or potentiation (LTP), to construct ensembles of highly linked neurons that encode a particular event or episode. But not nearly every event that is encountered is remembered, thus, a filter makes sure that only the most noteworthy ones are committed to memory. However, what does salience mean in this situation? According to one theory, salience is shown when an expected and seen result differs, creating a state of ambiguity that can be lessened by refreshing the pertinent memories.^103^ According to this theory, uncertainty causes neuromodulators to be released, which serve as a neurobiological filter governing which memories are created and modified. Interestingly, the increase in uncertainty and release of neuromodulators often originates from, and therefore occurs after, the salient event.

### 7.1 Dopamine‐mediated LTP in hippocampus

By engaging gene transcription and protein translation, dopamine can also promote LTP and LTD when the receptors are triggered following a plasticity‐inducing event **(**typically with a significant delay).^104^ According to the suggested mechanism, simultaneous stimulation causes translated proteins to be translocated to synapses that are ready for plasticity.^105^ Recent studies have suggested that dopamine may also contribute to the long‐term modulation of inhibition in CA3, which is important for memory consolidation.^106^ This regulation combines the delayed impact of dopamine with changes to the inhibitory network. These methods are appealing because they match the biological mechanisms of synaptic plasticity, which determine reward, novelty, and salience after the fact, with the timelines of behavioral learning.

### 7.2 Theta phase synchronization Between hippocampus and PFC

It is commonly known that the hippocampus and PFC synchronize more when behavioral demands are met. It is more difficult to assess how relevant such synchronicity is to behavior. The idea that the activation of prefrontal and hippocampus neurons aids in the information transfer, which is essential for the performance of tasks, is an appealing one. For instance, the hippocampus may send information regarding reward contingency and spatial position to the PFC's decision‐making machinery during spatial working memory tasks. This theory is supported by the finding that at the time of error trials in working memory tasks, PFC unit phase‐locking to the hippocampus theta is decreased.^67^ Showing that neurons with working memory that are associated with firing patterns are preferentially phase‐locked to the hippocampus theta rhythms (as opposed to those having no such firing associated with the task) would provide more concrete evidence for this theory. The authors showed a rough association between task‐related activity in PFC observed at the time of rewarded exploration and hippocampus phase‐locking, but there is currently no convincing evidence for such a relationship. Similarly, in a Y‐maze test (non‐working dependent memory),^88^ demonstrated that the coherence of hippocampal‐PFC theta peaks at the point of choice, supporting the idea that synchronization plays a part in directing choice behavior. The correlation of individual disparity in synchronization and behavior in animals has also been used to demonstrate relevance to behavior. For instance, learning‐impaired mutant mice's acquisition time for a spatial working memory test was predicted by the amount of theta‐frequency coherence between the hippocampus and PFC.^107^

### 7.3 Neuroplastic and dendritic spine growth and synaptic remodeling driven by enriched environments

Long‐lasting functional and structural alterations in neuronal circuits are caused by synaptic plasticity, and contextual enrichment can significantly improve this experience‐dependent remodeling. The molecular mechanisms that underlie learning cause spines to develop, vanish, or alter; they can even “remember” prior sensory experiences. Similar to LTP protocols, learning has also been shown to cause structural and functional changes in synapses.^108^ According to this learning theory, the stability of the underlying synaptic connections is crucial for memory persistence. However, synaptic structures are intrinsically very volatile, causing synaptic connections to continuously and spontaneously remodel without any activity;^109^ for instance, intact synapses can form even when the release from the pre‐synaptic terminal is eliminated^110^ or network silencing is eliminated.^111^

## 8 CHALLENGES, CONSIDERATIONS, AND FUTURE DIRECTIONS

Despite the fact that the existing evidence suggests the growing interest in using VR‐based cognitive enhancement on the aging population, several limitations limit the evidence. A good proportion of the literature makes conclusions about the permanency of cognitive improvements or generalization to older adults of varied sensory, motor, and cognitive profiles using small and non‐representative samples and brief interventions. Methodologically, the study of VR task designs, which are not usually standardized, makes it impossible to compare the results of the research in terms of novelty exposure, difficulty adaptation, or degree of immersion between the protocols. Inclusion of EEG in VR machines is one of the most important challenges in practice: motion artifacts, pressure of the headsets on electrodes, and electromagnetic interference have a severe adverse impact on signal quality, especially when using it in a mobile context or a highly interactive one. Also, adaptive intervention computational demands of real‐time signal processing are not yet straightforward and not all existing systems are able to extract oscillatory biomarkers (such as theta rhythms) of naturalistic VR behavior reliably.

Also, the variations in VR systems and technology make it more difficult to compare the findings of several VR‐based neurofeedback training (NFT). Some research used 3D monitors,^112^, ^113^ or even laptop screen;^114^ however, the majority of studies use head‐mounted display systems.^115^ In addition to affecting participants' behavior, performance, and brain activation, 3D VR systems with a stereoscopic stimulus presentation may be more immersive than screen systems with a monoscopic view, resulting in differences in the presence experience in VR.^116^ Individual variations in the dopamine drop present another difficulty. Furthermore, using VR as a feedback modality may not just be beneficial. Investigating and disclosing any potential negative impacts is crucial, as is identifying appropriate target audiences and NFT user groups for whom VR‐based feedback may not be helpful.

In the realm of VR, cybersickness is a well‐known problem that refers to the appearance of motion‐sickness‐like symptoms while interacting with virtual surroundings. Dizziness, nausea, oculomotor issues, and disorientation are possible symptoms. EEG‐VR with both systems may also make older adults feel discomfort, reducing compliance, fatigue or cybersickness that consequently results in the alteration of neurophysiological data. According to studies, up to 80% of VR users may experience some form of impact.^117^

Many individuals now have a VR system at home and use it mostly for entertainment because the market for reasonably priced VR headsets is expanding. People under 50 have the most familiarity with VR, whereas those over 50 have very little knowledge of the technology.^118^ When employing VR‐based NF, for example, with older people, this age difference needs to be taken into account. Research typically indicates that older users behave differently when navigating virtual worlds, experiencing varied levels of immersion and presence.^119^ Additionally, those over 40 are more likely to experience cybersickness symptoms.^120^ This could be because older people use technology less than younger people do. Therefore, the impact of VR‐based NFT may vary depending on the consumers' age. Additionally, there is a moral dilemma with neuroadaptive therapies. Pharmacological dopamine modulation and VR novelty exposure must be combined. Additionally, longitudinal studies and multi‐modal stimulation (VR + transcranial alternating current stimulation at theta frequency) should be conducted to examine the neuroprotective effects of VR novelty exposure against neurodegenerative diseases.

The personalization of novelty dose in VR‐based environment will be a priority in the future in order to optimize cognitive engagement, without causing stress and habituation.

Adaptive algorithms that dynamically titrate novelty intensity, frequency, and contextual uncertainty based on individual learning curves, age‐related neural variability, and moment‐to‐moment brain states will be critical for maximizing dopaminergic responsiveness and theta‐mediated plasticity in aging populations.

Another important direction is the multimodal incorporation of pupillometry, heart rate variability (HRV), as well as skin conductance into EEG to form a more detailed, real‐time measure of neuromodulatory condition. Closely related to arousal and locus coeruleusnorepinephrine (LCNE) activation, these peripheral physiological markers can be combined with theta oscillatory activity to permit closed‐loop VR interventions, which can infer motivational salience, cognitive effort, and fatigue more accurately than with EEG alone.

Lastly, the cooperative roles of the LC and VTA in VR‐based exploration should be directly assessed in the future. The integration of multimodal physiology and state‐of‐the‐art source‐resolved EEG and computational modeling will enable the dissociation of noradrenergic‐induced arousal with dopaminergic novelty signaling that will help understand how the LC‐VTA interaction is involved in the regulation of exploration, memory encoding, and adaptive behavior during aging. These findings would shape future VR interventions to use coordinated neuromodulatory mechanisms to maintain cognitive resilience throughout life.

## 9 CONCLUSION

Novelty has several facets. In addition to being new, a novel environment is inherently unpredictable because nothing that has never been experienced can be predicted. However, in daily life, people frequently attempt to lessen the surprise of going somewhere new, for instance, by looking at maps or photos beforehand. In animal studies examining the impact of novelty on memory, novelty and unexpectedness are frequently confused since such expectations are difficult to elicit in rodents or primates. Studies on event‐related potential have demonstrated that the psychophysiological reactions typically linked to novelty processing are primarily triggered by the unexpectedness of novelty. Any impacts of novelty on memory may be due to the unexpectedness of novel situations rather than novelty itself, since a breach of expectations might improve recall. Therefore, it is imperative that future human investigations disentangle the impacts of novelty and expectations that have been broken. Examining a new versus familiar environment may also have an impact on the brain and behavior by making decision‐making more difficult. It's possible that the positive effects of novelty on memory are due to active behavioral choices made in a novel versus familiar environment rather than the perceptual novelty of the environment itself. Therefore, the impacts of volition should also be included in future research examining the effects of spatial novelty as opposed to familiarity. By incorporating both active exploration and passive exposure situations, this may be objectively studied.

The histories of studying novelty in humans and animals are highly different, but as neuroscientific techniques have advanced, it has become simpler to transcend these distinctions and create connections between the two domains. The effective integration of smartphones with dependable mobile EEG equipment will enable the application of fresh research in human subjects. While individuals are actively exploring unfamiliar versus familiar locations, EEG could be recorded.

For instance, an experimental (memory) task could be presented via a connected smartphone. Many intriguing questions could be addressed using this method. For instance, is it true that exploring new places causes a theta state, and if so, how does this connect to the positive memory‐enhancing benefits of novelty? While bringing cognitive neuroscience research to the actual world may be its ultimate goal, it also raises new problems. There are currently no mobile neuroimaging techniques, and leaving the lab will raise the likelihood of unforeseen events, introduce noise, and make it more difficult to control for pertinent variables (like the level of novelty or familiarity of the surroundings). The neurological processes behind how spatial novelty affects learning and memory may be further clarified by pharmacological treatments in people. Expanding on the research on animals, it would be especially pertinent to discuss the role of the dopaminergic system, which is linked to memory and novelty processing. The development of reasonably priced, realistic, immersive 3D VR systems makes it possible to examine the impacts of spatial novelty while keeping a high degree of experimental control. Participants can explore controlled situations in the lab by employing virtual environments instead of real ones. Additionally, VR enables the creation of environments that are comparable in terms of size, number of landmarks, and other potentially pertinent elements. While it might not be feasible to have participants explore similar, familiar, and unfamiliar places in the real world, VR makes it simple to manage novelty and familiarity because prior exposure can be readily verified. Future studies can use smartphones, mobile EEG devices, or a mix of VR and neuroimaging approaches to examine the neurological underpinnings of exploring new worlds and the neuronal process underlying the effects of novelty exploration on human learning. Studies conducted on humans will enable the control of potential confounding variables, such as the influence of explicit expectations and volition, that are impossible to regulate in animals. When creating novelty‐exposure interventions that may be able to prevent or reduce age‐related memory loss, it will be extremely beneficial to have a deeper understanding of the precise elements of exploring a new environment that foster learning, as well as insight into the neurological underpinnings of this phenomenon.

## AUTHOR CONTRIBUTIONS

Ayodeji Johnson Ajibare developed the first idea for the study and Abraham Olufemi Asuku wrote the first draft of the manuscript. Abraham Olufemi Asuku and Ayodeji Johnson Ajibare contributed to the study's concept, literature search, figure design, editing, reviewing, and approval of the manuscript's final draft.

## CONFLICT OF INTEREST STATEMENT

The authors declare no conflicts of interest.

## ETHICS STATEMENT

Not applicable.

## Data Availability

Data sharing is not applicable to this article as no datasets were generated or analyzed during the current study.
